# Green Tea Polyphenols Induce p53-Dependent and p53-Independent Apoptosis in Prostate Cancer Cells through Two Distinct Mechanisms

**DOI:** 10.1371/journal.pone.0052572

**Published:** 2012-12-20

**Authors:** Karishma Gupta, Vijay S. Thakur, Natarajan Bhaskaran, Akbar Nawab, Melissa A. Babcook, Mark W. Jackson, Sanjay Gupta

**Affiliations:** 1 Department of Urology, Case Western Reserve University, Cleveland, Ohio, United States of America; 2 Department of Nutrition, Case Western Reserve University, Cleveland, Ohio, United States of America; 3 Department of Pathology, Case Western Reserve University, Cleveland, Ohio, United States of America; 4 University Hospitals Case Medical Center, Cleveland, Ohio, United States of America; 5 Case Comprehensive Cancer Center, Cleveland, Ohio, United States of America; Roswell Park Cancer Institute, United States of America

## Abstract

Inactivation of the tumor suppressor gene p53 is commonly observed in human prostate cancer and is associated with therapeutic resistance. We have previously demonstrated that green tea polyphenols (GTP) induce apoptosis in prostate cancer cells irrespective of p53 status. However, the molecular mechanisms underlying these observations remain elusive. Here we investigated the mechanisms of GTP-induced apoptosis in human prostate cancer LNCaP cells stably-transfected with short hairpin-RNA against p53 (LNCaPshp53) and control vector (LNCaPshV). GTP treatment induced p53 stabilization and activation of downstream targets p21/waf1 and Bax in a dose-dependent manner specifically in LNCaPshV cells. However, GTP-induced FAS upregulation through activation of c-jun-N-terminal kinase resulted in FADD phosphorylation, caspase-8 activation and truncation of BID, leading to apoptosis in both LNCaPshV and LNCaPshp53 cells. In parallel, treatment of cells with GTP resulted in inhibition of survival pathway, mediated by Akt deactivation and loss of BAD phosphorylation more prominently in LNCaPshp53 cells. These distinct routes of cell death converged to a common pathway, leading to loss of mitochondrial transmembrane potential, cytochrome c release and activation of terminal caspases, resulting in PARP-cleavage. GTP-induced apoptosis was attenuated with JNK inhibitor, SP600125 in both cell lines; whereas PI3K-Akt inhibitor, LY294002 resulted in increased cell death prominently in LNCaPshp53 cells, establishing the role of two distinct pathways of GTP-mediated apoptosis. Furthermore, GTP exposure resulted in inhibition of class I HDAC protein, accumulation of acetylated histone-H3 in total cellular chromatin, resulting in increased accessibility of transcription factors to bind with the promoter sequences of p21/waf1 and Bax, regardless of the p53 status of cells, consistent with effects elicited by an HDAC inhibitor, trichostatin A. These results demonstrate that GTP induces prostate cancer cell death by two distinct mechanisms regardless of p53 status, thus identifying specific well-defined molecular mechanisms that may be targeted by chemopreventive and/or therapeutic strategies.

## Introduction

With limited treatment options available for prostate cancer, patients with relapsing disease are treated with anti-androgens. However, initial clinical response is often followed by the emergence of hormone-refractory and eventually chemotherapy-resistant cancer [1]. It is well established that cancer cells may acquire chemoresistance through a variety of mechanisms, most of them implying an altered apoptotic program [2]. Recent studies have demonstrated that p53 status might be a critical determinant for chemo-sensitivity in human tumors [3], [4]. More than 50% of human cancers, including prostate cancer, exhibit loss of normal p53 functions and/or defects in the p53 signaling pathway as well as missense mutations or deletions; these molecular alterations are associated with resistance to cell death [4], [5]. The relative ineffectiveness of current chemotherapeutic regimens justifies a continued search for safe and effective agents that might improve treatment and/or inhibit the development of resistance to chemotherapy.

The p53 protein, a tumor suppressor, functions in the transcription of growth inhibiting genes involved in apoptosis, cell cycle arrest and DNA repair [4]–[6]. The tumor suppressive function of p53 is mainly attributed to its role in one of two mechanisms: either promoting the repair and survival of damaged cells, or promoting the permanent removal of irreparably damaged cells through apoptosis [6], [7]. For example, p53 causes cell cycle arrest primarily by activating the transcription of a cyclin-dependent kinase inhibitor, p21/waf1, and induces apoptosis via transcriptional activation of the pro-apoptotic Bcl2 family genes, Bax, PUMA and Noxa [7]. An alternative and complementary signaling pathway that leads to programmed cell death includes the extrinsic death receptor pathway. The extrinsic pathway is initiated upon receptor ligation of FAS/CD95 ligand mediated by an adapter molecule FAS-associated death domain (FADD) that bridges the receptor with the downstream effector, caspase 8, resulting in the assembly of the death-inducing signaling complex [7], [8]. The extrinsic and intrinsic apoptosis pathways are connected by the caspase-8-mediated cleavage of the pro-apoptotic Bcl-2 family member Bid. Truncated Bid (tBid) translocates to mitochondria, where it induces the release of cytochrome C, followed by induction of apoptosis [9].

As deregulation of the p53 pathway in a cancer cell is a common event and may contribute to drug resistance, chemotherapeutic strategies aimed at this defective mechanism are needed. For example, a new therapeutic approach, involving pharmacological inhibition of histone deacetylases (HDACs), allows local remodeling of chromatin and dynamic changes in the nucleosomal packaging, via acetylation/de-acetylation of core histone protein, and thus plays a pivotal role in the regulation of accessibility to chromosomal DNA, and thereby, in the regulation of gene transcription [10]. Among the most important regulators of such phenomena are specific enzymes that regulate N-terminal acetylation of lysine residues on H3 and H4 histones, the histone acetyltransferases (HATs) and HDACs [10], [11]. These enzymes can be recruited to modify specific genes in complexes by sequence-specific transcription factors. Inhibition of HDAC activity causes cell cycle arrest and apoptosis in cancer cells, primarily through transcriptional activation of p53-mediated pro-apoptotic response and induction of cell cycle kinase inhibitor p21/waf1 and Bax, as well as through transcriptionally-independent direct binding of p53 to Bax, Bcl2 and Bcl-_xL_ [12], [13].

Next to inactivation of apoptotic signaling elements tumors also develop chemotherapy resistance by activation of survival signaling such as the phosphatidylinositide 3-kinase (PI3K)/Akt pathway [14]. Activation of PI3K by receptor tyrosine kinases leads to the recruitment of the protein kinase B (PKB/Akt) to the plasma membrane where it is subsequently activated upon phosphorylation at residues Thr308 and Ser473, which are in turn phosphorylated by phosphoinositide-dependent kinases. Activated Akt enhances the survival of cells both by the inhibition of pro-apoptotic proteins *viz*. BAD or caspase-9 and by activation of anti-apoptotic proteins thereby promoting cell survival [14], [15].

In the past few decades, a number of natural polyphenolic compounds have been evaluated for their possible use in the prevention and treatment of cancer [16]. Green tea polyphenols, the major constituent of which is epigallocatechic-3-gallate (EGCG), have been shown to induce cell cycle arrest and apoptosis in various cancer cell types [17]. Our laboratory has conducted extensive investigations of the mechanisms underlying the anti-carcinogenic effects of green tea polyphenols in human prostate cancer cells [12], [13]. We previously demonstrated that green tea polyphenols cause apoptosis in prostate cancer cells irrespective of androgen association and p53 status [13]. Furthermore, we demonstrated that GTP and EGCG increase p53 transcriptional activity and acetylation by suppressing class I histone deacetylases [12]. In the present study, our results demonstrate that green tea polyphenols induce apoptosis in prostate cancer cells by activating the FAS death receptor/caspase-8 pathway and inhibiting the p-Akt/p-BAD cell survival pathway. Our accumulated findings have important implications for our understanding of the molecular mechanism of chemoresistance in prostate cancer cells, and in particular, the role of p53 and Akt in this process. Since chemoresistance is a limiting factor in the efforts to provide successful treatment for prostate cancer, it is critical to understand how GTP differentially overcomes therapeutic resistance and induces apoptosis in prostate cancer cells with varying types of p53 abnormalities.

## Materials And Methods

### Cell Culture and Reagents

LNCaPshV and LNCaPshp53 cells were generated by infecting human prostate cancer LNCaP cells obtained from American Type Culture Collection (Manassas, VA) with shp53 lentivirus prepared in the laboratory by transfecting 293T cells with lentiviral vectors pLVTHSiGFP or pLVTHMshp53RNA and packaging constructs as previously described [18], [19]. Cells were grown and maintained in RPMI 1640 media (Hyclone, Thermo Fischer, Logan, UT) supplemented with 1% penicillin-streptomycin and 10% fetal bovine serum (Foundation, West Sacramento, CA) at 50–70% confluence. Cells received the following treatments: 20 ng/ml trichostatin A (Sigma, St Louis, MO), dissolved in DMSO; 20–80 µg/ml Polyphenon E® (Mitsui Norin, Japan) hereafter referred as green tea polyphenols (GTP) for indicated times. Concentrations of 10 µg/ml Polyphenon E correspond to 14 µM EGCG as determined by HPLC analysis. The constituents present in Polyphenon E® are previously described [20]. Antibodies for anti-p53 (SC-126), anti-p21/waf1 (SC-397), anti-Akt (SC-8312), anti-Bax (SC-493), anti-BID (SC-6538), anti-HDAC1 (SC-7872), anti-HDAC2 (SC-6296), anti-HDAC3 (SC-11417), anti-HDAC8 (SC-11405), anti-FAS (SC-715), anti-FADD (SC-5559), anti-p-FADD Ser194 (SC-12439), anti-caspase-8 (SC-7890) and anti-β-actin (SC-47778) were purchased from Santa Cruz Biotechnology (Santa Cruz, CA). Antibodies for anti-histone H3 (05-928), anti-histone acetyl H3 Lys9/18 (07-593) from Upstate (EMD Millipore, Billerica, MA), and BID (44-4334) was procured from Invitrogen Corporation (Camarillo, CA). Antibodies for anti-caspase-9 (#9502), anti-caspase-3 (#9662), cleaved PARP (#9544), anti-c-IAP (#3130), anti-X-IAP (#2045), anti-SAPK/JNK (#9258), anti-p-JNK Thr183/Tyr185 (#9251), anti-p-Akt Ser473 (#4051), anti-BAD (#9292), anti-p-BAD Ser136 (#9295) and anti-VDAC (#4866) were purchased from Cell Signaling Technologies (Danvers, MA).

### Generation of LNCaPshV and LNCaPshp53 Cells

293T packaging cells were plated in 100-mm plates the day before transfection in DMEM containing 10% heat inactivated FBS without penicillin–streptomycin. The cells were transfected with 6 µg of shp53 or shGFP (control) RNA along with second generation packaging constructs (pCMV-dR8.74 and pMD2G) using lipofectamin Plus reagent (Invitrogen Corp.) as per the protocol provided by the vendor. For two subsequent days, media was collected and layered onto the LNCaP cells after adding 10 µl of 4 mg/ml polybrene per 10 ml and sterilize through filtering.

### Proliferation Assay

The effect of GTP on cell proliferation was determined by MTT [3-(4, 5-dimethyl-thiazol-2-yl)-2, 5-diphenyl tetrazoliumbromide] assay and growth inhibition was assessed as the percent viability where vehicle-treated cells were taken as 100% viable as previously described [21].

### Methylene Blue Staining and Quantification

Cells were plated in 6 well culture plates and treated with various concentration of camptothecin (50–200 ng/ml) for 24 h. Media was then removed and cells fixed with methylene blue solution after treatment and plates were kept on shaker for 1 h. The plates were gently washed with double-distilled water to remove excess dye, dried and scanned.

### Light Microscopy

LNCaPshV and LNCaPshp53 cells were grown to 70% confluence and treated with 20–40 µg/ml concentration of GTP for 24 h. The photographs were captured at x40 magnification using light microscope.

### DNA Fragmentation Assay

The cells were grown to approximately 70% confluence and treated with 40 µg/ml concentration of GTP for 48 h. Cells were then subjected to processing for DNA isolation and fragmentation assay. The bands were visualized under a UV transilluminator, followed by digital photography as previously described [21].

### Cell Death Assay

Cell apoptosis was measured by *in vitro* determination of cytoplasmic histone-associated-DNA-fragments (mono and oligonucleosomes) after induction of cell death by photometric enzyme immunoassay using Cell Death Detection ELISA kit from Roche (Cat#11774425001) as per vendor’s protocol. Briefly, cells were treated with 20 µM LY294002 or 20 µM SP600125 for 8 h and 40 µg/ml GTP for 16 h or in combination for 24 h and cytoplasmic cell extracts were transferred onto the streptavidin coated microwell plates with immuno-reagent containing anti-histone-biotin, anti-DNA-POD. After incubation, enzymatic reaction was performed and color was read at 405 nm using reference wavelength as 490 nm. Background values (incubation buffer alone) were subtracted, and OD values representing nucleosomal DNA fragments in treated samples were compared with those values obtained from untreated control cells, and expressed as fold increase.

### Western Blot Analysis

Cells were lysed in radioimmunoprecipitation assay (RIPA) buffer (containing 1% NP40, 0.5% sodium deoxycholate, 0.1% SDS in PBS, and freshly added complete protease inhibitor cocktail (Roche Applied Sciences, Indianapolis, IN). Protein concentration in the cell lysate was determined using detergent-compatible protein assay from Bio-Rad (Hercules, CA). Protein samples were subjected to SDS-PAGE and transferred to nitrocellulose membrane. The membrane was incubated with primary antibody for overnight blocked in 5% nonfat milk in PBS. Next day membrane was removed from primary antibody, washed with washing buffer and subsequently incubated with appropriate horseradish peroxidase (HRP)–conjugated secondary antibody for 1 h at room temperature. Membrane was then developed with enhanced chemiluminescence reagent (GE Healthcare, Piscataway, NJ) and exposed to Hyblot CL autoradiography film (Denville Scientific, Metuchen, NJ). Image digitization and quantification were performed with Kodak 2000 imaging system.

### Extraction and Expression of Acetylated Histone Proteins

Human prostate cancer LNCaPshV and LNCaPshp53 cells were treated with 20–80 µg/ml GTP for 24 h and were harvested then washed twice with ice-cold phosphate-buffered saline (PBS) supplemented with 5 mM sodium butyrate. After wash, cells were resuspended in Triton extraction buffer [PBS containing 0.5% Triton X-100 (vol/vol), 2 mM phenylmethylsulfonyl fluoride, 0.02%(wt/vol) NAN3] and lysed on ice for 10 min with gentle stirring, centrifuged at 2000 r.p.m. for 10 min at 4°C. Pellet was washed in Triton extraction buffer and then resuspended in 0.2 N HCl. Histones were acid extracted overnight at 4°C and centrifuged at 2000 rpm for 10 min at 4°C. Samples were processed for the analysis of histones using immunoblotting.

### Chromatin Immunoprecipitation (ChIP) Assay

Human prostate cancer LNCaPshV and LNCaPshp53 cells were treated with 20–80 µg/ml doses of GTP dissolved in PBS or only with PBS (control) for 3 days. At the end of treatment, cells were incubated in serum containing 1% formaldehyde for 15 min at room temperature for cross-linking. Reaction was then terminated using 0.125 M final concentration of glycine. The chromatin was digested with monococcal nuclease enzyme and incubated with anti-acetylated histone H3 (Cat#07-593; Upstate Biotechnology) antibody overnight at 4°C. Cross-linking was reversed by incubating the samples overnight at 65°C. DNA was purified using phenol-chloroform-isoamyl solution with extraction followed by ethanol precipitation. DNA was then resuspended in nuclease-free water. Primers used for p21/waf1 gene promoter were as follows: forward primers 5′-GTGGCTCTGATTGG CTTTCTG-3′, reverse primers 5′-GTGAAAACAGGCAGCCCAAG-3′ and for Bax gene promoter, forward primers 5′-TAATCCCAGCGCTTTGGAA-3′ and reverse primers 5′-TGCA GAGACCTGGATCTAGCAA-3′, respectively. Immunoprecipitated DNAs, beads or input controls were subjected to polymerase chain reaction (PCR) amplification for 30 cycles of the following cycling conditions: Stage-1–95°C for 2 min (1 cycle), Stage-2–95°C for 30 s, 60°C for 30 s, 72°C for 1 min (30 cycles), Stage-3–72°C for 3 min (1 cycle). PCR products were subjected to electrophoresis using 2% agarose gel.

### Cell Cycle Analysis and Apoptosis Detection

The effect of GTP on cell cycle and subG1 was measured by performing flow cytometric assay. LNCaPshV and LNCaPshp53 cells were treated with GTP and were harvested by trypsinization post-treatment (both attached and floating cells from post-treatment media) were collected. Cells were washed twice with PBS. Approximately, 1×10^6^ cells were fixed in 90% cold methanol and left on ice for at least 30 min and stored at –20°C till processed for cell cycle. Cells were pelleted, washed, and resuspended in 0.04 µg/ml propidium iodide and 100 µg/ml RNase in PBS. The samples were incubated at room temperature for 30 min and flow cytometry was performed on EPICS-XL MCL flow cytometer and analyzed using Cell Quest Analysis software Modfit to determine the number of cells in each phase of the cell cycle.

### Statistical Analysis

All experiments were repeated at least twice in duplicate. Results are expressed as mean values ± SD. The images were digitized and quantification was performed using a software program with Kodak 2000 imaging system. Statistical comparisons were performed by ANOVA followed by a Dunnett’s multiple comparison test. *P* values <0.05 were considered significant.

## Results

**Figure 1:**
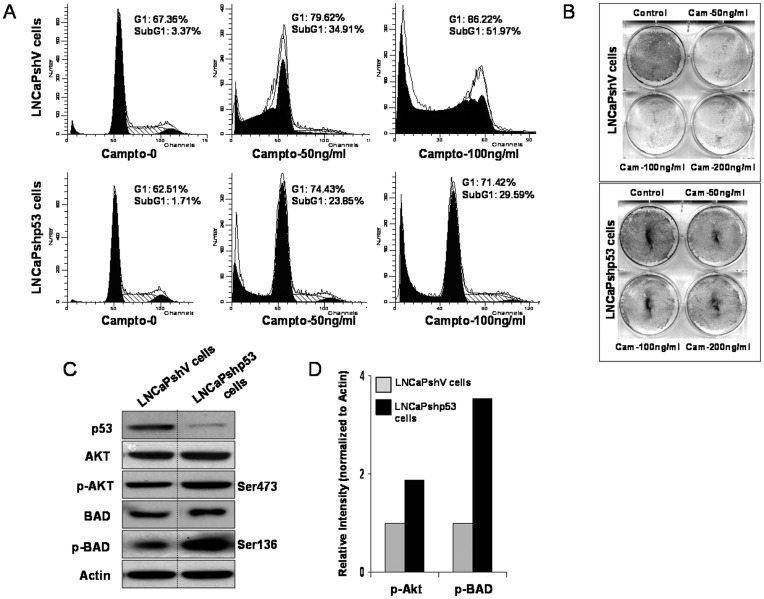
Camptothecin induces differential apoptosis in LNCaPshV and LNCaPshp53 cells. Isogenic cells with lenti-virus vector background were generated by permanent knockdown of p53 in LNCaP cells [LNCaPshp53] and transfected with control vector, LNCaPshV cells. [**A**] Cell treated with 50 and 100 ng/ml camptothecin for 24 h were harvested, stained with PI and analyzed by flow cytometry to measure sub-G1 and G1 population. [**B**] Cells were treated with 50, 100 and 200 ng/ml of camptothecin for 24 h and stained with methylene blue. The intensity of methylene blue taken up by live cells was measured spectrophotometerically after eluting the dye in 0.1N HCl and compared with untreated cells. [**C**] Knockdown of p53 upregulated Akt/BAD signaling in prostate cancer cells. LNCaPshV and LNCaPshp53 cells were lysed and Western blotting was performed for p53, Akt, p-Akt (Ser473), BAD, p-BAD (Ser136) proteins. Actin was used as internal loading control. [**D**] Relative intensities of p-Akt and p-Bad protein in LNCaPshV and LNCaPshp53RNA cells where bands were normalized to actin and expressed in relative values compared to the native protein. The details are described in the materials and methods section.

Although the efficacy of GTP in inducing apoptosis in a variety of cancer cell types has been well documented [22]–[24], the effect of GTP in the absence of p53 abnormalities has not been investigated in detail. Earlier studies have shown that inactivation of p53 by siRNA in human prostate cancer LNCaP cells increased resistance to EGCG-mediated apoptosis [25]. To unequivocally establish the role of p53 and to investigate pathways responsible for increased cellular resistance, we generated isogenic cells with lentivirus vector background by permanently knockdown p53 in LNCaP cells to generate LNCaPshp53 and transfected with control vector to generate LNCaPshV cells. To determine if p53 levels affect cell death response, the isogenic cells were treated with camptothechin, a DNA damaging anticancer agent which causes apoptosis in various human cancer cells [26]. As shown in **figure 1A**, treatment with 50 and 100 ng/ml camptothechin for 24 h resulted in significant increase of LNCaPshV cells in the G1 phase of the cell cycle. Compared to untreated control, the percentage of cells in G1 phase increased from 67.36% to 79.62% and 86.22% after treatment with 50 and 100 ng/ml concentrations of camptothecin. Likewise, LNCaPshp53 cells exhibited G1 phase arrest after camptothecin treatment with 74.43% and 71.42% after treatment with 50 and 100 ng/ml camptothecin, compared to untreated cells with 62.51% in the G1 phase. In addition to G1 phase arrest, camptothecin also caused significant accumulation of cells in subG1 phase, indicative of apoptosis. Compared to untreated cells (3.37%), treatment of LNCaPshV cells with camptothecin increased from 34.91% at 50 ng/ml and 51.97% at 100 ng/ml in the sub-G1 phase. However, LNCaPshp53 cells were more resistant to camptothechin-mediated apoptosis with 23.87% and 29.59% at 50 ng/ml and 100 ng/ml doses in the subG1 phase, compared to 1.71% in the untreated controls. Similar results were noted in the clonogenic assay where knockdown of p53 resulted in increased resistance to camptothechin-mediated cell death (**Figure 1B**).

### Knockdown of p53 Increases Akt Survival Signaling Pathway

Since studies have shown that activated Akt can lead to downregulation of p53 levels [27], we next determined the effect of p53 knockdown on Akt levels and its downstream targets. As shown in **figure 1C**, knockdown of p53 caused a significant increase in p-Akt (Ser473) and p-BAD (Ser136) expression in LNCaPshp53 cells, compared to LNCaPshV cells. A 2.0 fold increase in p-Akt levels and 3.78 fold increase in p-BAD levels were noted after p53 knockdown in LNCaPshp53 cells, compared to LNCaPshV cells (**Figure 1D**).

### Green Tea Polyphenols Induce Apoptosis in Both LNCaPshV and LNCaPshp53 Cells Independent of p53 status

**Figure 2:**
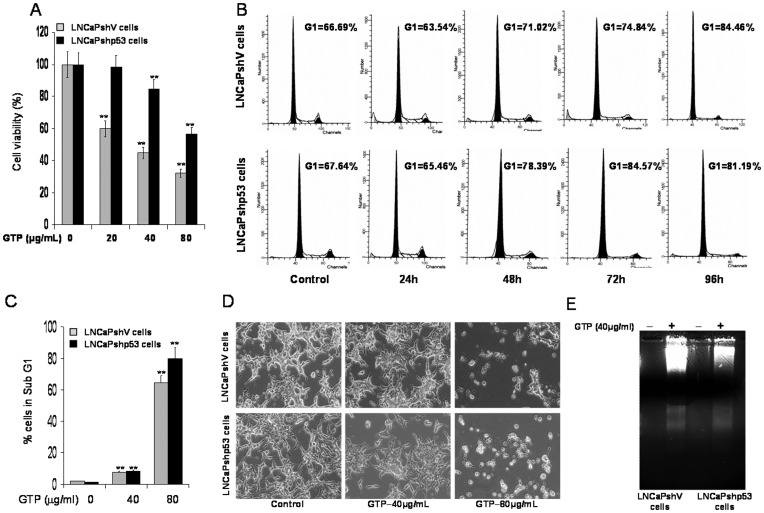
Green tea polyphenols decrease cell viability and induces apoptosis in LNCaPshV and LNCaPshp53 cells. [**A**] Cells were exposed to 20–80 µg/ml concentration of GTP for 24 h, and viability of the cells was determined by the MTT assay. Cell viabilities are depicted as percentages; vehicle-treated cells were regarded as 100% viable. The bars represent mean±SD of at least two independent experiments each performed in duplicate, **p<0.001 represents significant differences as compared to control group without GTP treatment. [**B**] Cell were treated with 40 µg/ml GTP for 24, 48, 72 and 96 h and distribution of cells were recorded in different stages of cell cycle analyzed using FACS analysis. [**C**] Cells were treated with 40 and 80 µg/ml GTP for 96 h and the number of cells undergoing apoptosis were determined by measuring cell population in sub G1 phase of the cell cycle. The bars represent mean±SD of at least two independent experiments each performed in duplicate, **p<0.001 represents significant differences as compared to control group without GTP treatment. [**D**] Light microscopic images of LNCaPshV and LNCaPshp53 cells treated with 40 and 80 µg/ml GTP for 96 h. GTP treatment exhibits morphological changes consistent with apoptosis in both these cells. [**E**] DNA fragmentation assay. The cells were treated with 40 µg/ml concentration of GTP for 48 h, collected for DNA isolation and subjected to agarose gel electrophoresis, followed by visualization of bands under UV light. The details are described in the materials and methods section.

To characterize p53 status on the effect of GTP treatment, subsequent studies were performed in LNCaPshV and LNCaPshp53 cells. As shown in **figure 2A**, MTT assay performed after 24 h of treatment with 20–80 µg/ml concentration of GTP exhibited dose-dependent inhibition in cell viability from 100% to 33.98% in LNCaPshV and 100% to 66% in LNCaPshp53 cells. In comparison to LNCaPshp53 cells, LNCaPshV cells were more sensitive to GTP exposure in the initial 24 h of treatment. To study the effects of more prolonged exposure to GTP, time-dependent studies were performed with 40 µg/ml dose of GTP. Treatment of cells with GTP caused marked accumulation of cells in G0/G1 phase; 71.02% to 84.57% in LNCaPshV cells, compared to 78.39% to 81.19% in LNCaPshp53 cells between 48–96 h, respectively (**Figure 2B**). The number of cells in subG1 also increased significantly at 96 h post-GTP treatment in both cells lines; 0.53% to 66.97% in LNCaPshV cells and 0.31% to 83.94% in LNCaPshp53 cells, regardless of their p53 status (**Figure 2C**). GTP treatment also resulted in apoptosis of both LNCaPshV and LNCaPshp53 cells as observed by light microscopy and DNA fragmentation assay (**Figure 2** **D&E**).

**Figure 3:**
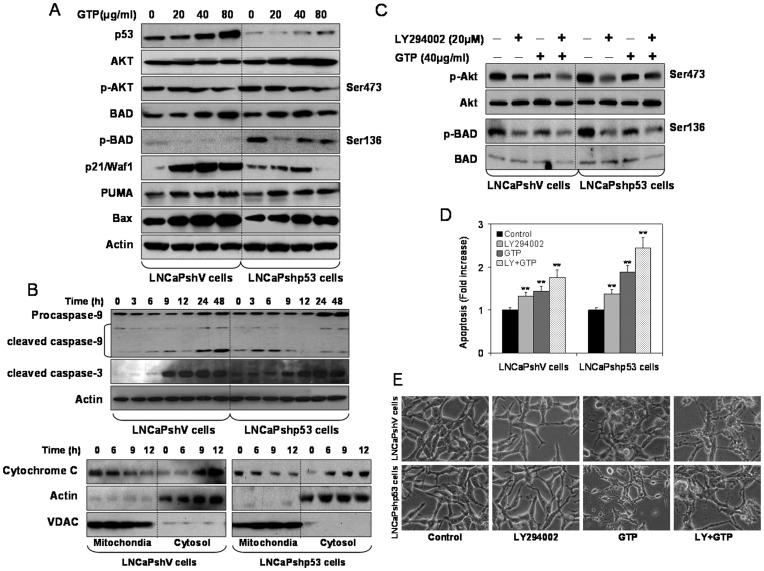
Green tea polyphenols induce apoptosis irrespective of p53 status in prostate cancer LNCaPshV and LNCaPshp53 cells. [**A**] Cells were treated with 20–80 µg/ml concentration of GTP for 24 h and Western blotting was performed for p53, Akt, p-Akt (Ser473), BAD, p-BAD (Ser136), p21/waf1, PUMA and Bax proteins. [**B**] Cells were treated with 40 µg/ml concentration of GTP for 3, 6, 9, 12, 24 and 48 h and Western blotting was performed for procaspase-9, cleaved caspase-9 and cleaved caspase-3 proteins. A typical actin blot demonstrates internal loading control. Cytochrome c release from mitochondria to cytosol was determined by Western blotting in GTP treated cells. A typical actin blot demonstrates internal loading control for cytosol whereas VDAC as internal loading control for mitochondria. [**D**] Cells were treated with 20 µM concentration of PI3K-Akt inhibitor LY294002 for 8 h and with 40 µg/ml GTP for 16 h alone, or LY294002 for 8 h followed by GTP treatment in combination followed by Western blotting for p-Akt, Akt, p-BAD and BAD proteins. The expressions of native proteins were considered as loading controls. [**D**] Cell death measurement was performed by photometric enzyme immunoassay Cell Death Detection ELISA kit. The bars represent mean±SD of at least two independent experiments each performed in duplicate, **p<0.001 represents significant differences as compared to control group. [**E**] Light microscopic images of LNCaPshV and LNCaPshp53 cells treated with LY294002 and GTP alone or in combination. The details are described in the materials and methods section.

Previously we reported that in human prostate cancer cells containing functional p53, GTP constituent EGCG treatment upregulates p53 and its phosphorylation on critical serine residues and p14^ARF^-mediated downregulation of MDM2 in p53-dependent manner [28]. Treatment of LNCaPshV cells with 20–80 µg/ml concentrations of GTP for 24 h exhibited a dose-dependent increase in p21/waf1, Bax and PUMA, well known downstream targets of p53, whereas no significant alterations were noted in p-BAD and p-Akt levels. In LNCaPshp53 cells, GTP treatment also resulted in a dose-dependent increase in the expression of p21/waf1, Bax and PUMA, although not to the extent observed in LNCaPshV cells, indicating both p53-dependent and p53-independent induction of pro-apoptotic proteins in these cells (**Figure 3A**). Following treatment with GTP a significant decrease in Akt and BAD phosphorylation was evident in LNCaPshp53 cells. Consistent with apoptosis, time-dependent increases in cleaved caspase 9 and caspase 3 were observed in both cell lines (**Figure 3B**).

Next, we analyzed the effects of GTP exposure on the downstream apoptotic pathway, specifically, the effectors of the mitochondrial death cascade, by evaluating cytochrome C release in the cytosol. Treatment of LNCaPshV and LNCaPshp53 cells with GTP significantly increased cytochrome C levels in the cytosol which peaked at 12 h post-GTP exposure. Translocation of cytochrome C from mitochondria to cytosol was observed in both cell lines in a time-dependent manner (**Figure 3B**).

### Green Tea Polyphenols Inhibit Akt Ser473 Phosphorylation and Downstream Targets in LNCaPshp53 Cells

Earlier we demonstrated that p53 knockdown resulted in increased expression of Akt and increased its phosphorylation at Ser473. Next we treated both cell lines with GTP and/or LY294002, a specific PI3K-Akt inhibitor. Phosphorylation of Ser473 and T308 activates Akt to phosphorylate target proteins through its kinase activity to promote survival and inhibit apoptosis [29]. As shown in **figure 3C & D**, treatment of cells with LY249002 caused a reduction in p-Akt levels and resulted in increased apoptosis in both cell lines although the extent of apoptosis was higher in LNCaPshp53 cells. Similarly, GTP treatment inhibited Akt phosphorylation at Ser473, which was consistent with increased apoptosis, and combined treatment with GTP and LY294002 resulted in an even higher level of cell death in LNCaPshp53 cells than in LNCaPshV cells. Consistently, phosphorylation of BAD was decreased significantly as a result of decreased phosphorylation of kinase activity of Akt by LY294002 and GTP; this correlated with simultaneously increased cell death of LNCaPshp53 cells. Similar effects of lower magnitude were observed in LNCaPshV cells (**Figure 3** **D&E**). These results demonstrate that although the magnitude of cell death caused by GTP exposure is similar in both cell lines, the pathways that lead to apoptosis are different and might be influenced by p53 status.

### Green Tea Polyphenols Induce the Death Receptor Pathway in Human Prostate Cancer Cells

**Figure 4:**
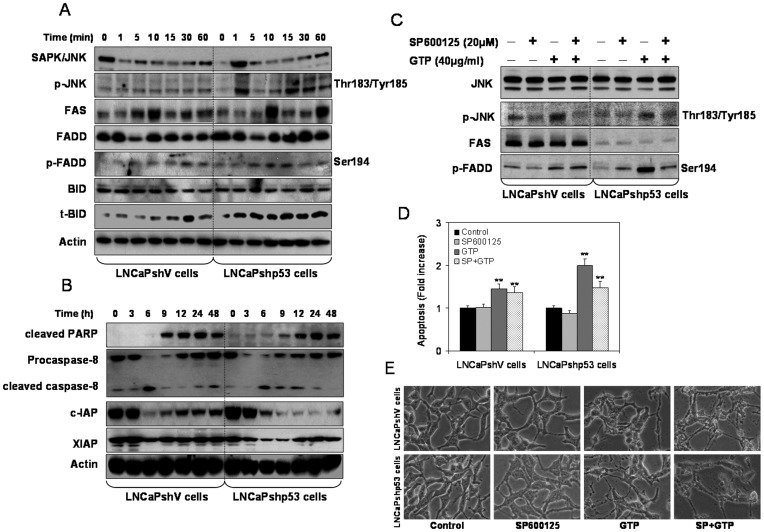
Green tea polyphenols-mediated apoptosis induced by death receptor pathway in prostate cancer LNCaPshV and LNCaPshp53 cells. [**A**] Cells were treated with 40 µg/ml concentration of GTP for 1, 5, 10, 15, 30 and 60 min and Western blotting was performed for SAPK/JNK, p-JNK, FAS, FADD, p-FADD, BID and t-BID proteins. A typical actin blot demonstrates internal loading control. [**B**] Cells were treated with 40 µg/ml concentration of GTP for 3, 6, 9, 12, 24 and 48 h and Western blotting was performed for cleaved PARP, procaspase-8, cleaved caspase-8, c-IAP and XIAP proteins. A typical actin blot demonstrates internal loading control. [**C**] Cells were treated with 20 µM concentration of JNK inhibitor SP600125 for 8 h and with 40 µg/ml GTP for 16 h alone, or SP600125 for 8 h followed by GTP treatment in combination followed by Western blotting for JNK, p-JNK, FAS and p-FADD proteins. The expression of native JNK protein was considered as loading control. [**D**] Cell death measurement was performed by photometric enzyme immunoassay Cell Death Detection ELISA kit. The bars represent mean±SD of at least two independent experiments each performed in duplicate, **p<0.001 represents significant differences as compared to control group. [**E**] Light microscopic images of LNCaPshV and LNCaPshp53 cells treated with SP600125 and GTP alone or in combination. The details are described in the materials and methods section.

In order to explore possible mechanisms involved in GTP-induced apoptosis, we next focused on the extrinsic pathway through FAS. As shown in **figure 4A**, treatment with GTP caused induction of FAS within 10 min in both cell lines, followed by increased levels of phosphorylated FADD at Ser194, whereas total FADD levels remained unchanged. However, the basal levels of FADD were much higher in LNCaPshp53 cells as compared to LNCaPshV cells. Searching for the upstream effectors of FAS upregulation revealed that JNK was activated by GTP, as was evident from the increase in p-JNK status. The increase in p-JNK (Thr183/Tyr185) was more pronounced in LNCaPshp53 cells, compared to LNCaPshV cells. The involvement of JNK in GTP-induced FAS upregulation was further confirmed by the use of JNK inhibitor SP600125. GTP at 40 µg/ml phosphorylated JNK and FADD at Ser194, and FADD phosphorylation was inhibited by the JNK inhibitor, SP600125 (**Figure 4C**). The cell death data further confirmed that inhibition of JNK resulted in reduced apoptosis in both LNCaPshV and LNCaPshp53 cells, and the extent of apoptosis was independent of p53 status in these cells (**Figure 4** **D&E**). This was further confirmed by the experiment in which pre-treatment with FAS neutralizing antibody abrogated GTP-induced apoptosis almost to the same extent in both cell lines (data not shown). These results support our hypothesis that GTP-induced apoptosis in cancer cells is activated by the FAS death receptor/caspase 8 pathway. Furthermore, treatment of LNCaPshV and LNCaPshp53 cells with GTP caused cleavage of caspase-8 as early as 6 h after treatment in both cell lines, with concomitant reductions in the inhibitors of apoptosis c-IAP and XIAP (**Figure 4** **A&B**). Since cleavage of Bid is achieved through the caspase-8 pathway, we next determined the expression of t-Bid upon GTP treatment. As shown in **figure 4A**, exposure of cells to GTP resulted in gradual accumulation of t-Bid in both LNCaPshV and LNCaPshp53 cells, suggesting that the extrinsic pathway mediates GTP-induced apoptosis in prostate cancer cells, regardless of their p53 status.

### Green Tea Polyphenols Induce Apoptosis Independently through Intrinsic and Death Receptor Pathway in Human Prostate Cancer Cells

After identifying the pathways involved in GTP-induced apoptosis of prostate cancer cells, we sought to determine i) whether these two pathways operate independently and ii) whether outcome varies according to p53 status. Previous results had shown that blocking the death receptor pathway by the use of either pharmacological inhibition of JNK by SP600125 or the use of FAS neutralizing antibody resulted in partial abrogation of GTP-mediated apoptotic effects, with no difference in the extent of apoptosis (**Figure 4** **C&D**). In the intrinsic pathway, GTP treatment caused apoptosis in LNCaPshV cells with marked increase in p21/waf1 Bax and PUMA, and in LNCaPshp53 cells caused significant decrease in Akt/BAD phosphorylation and release of Bax as a trigger for apoptosis (**Figure 3A**). These results convincingly prove that GTP-induced cell death involves both the extrinsic and the intrinsic pathway, which operate independently of one another in these cell lines. Divergent apoptosis was exhibited in the intrinsic pathway because of varying p53 status.

### Green Tea Polyphenols Decrease Class I Histone Deacetylases and Increase Histone Acetylation in Prostate Cancer Cells

**Figure 5:**
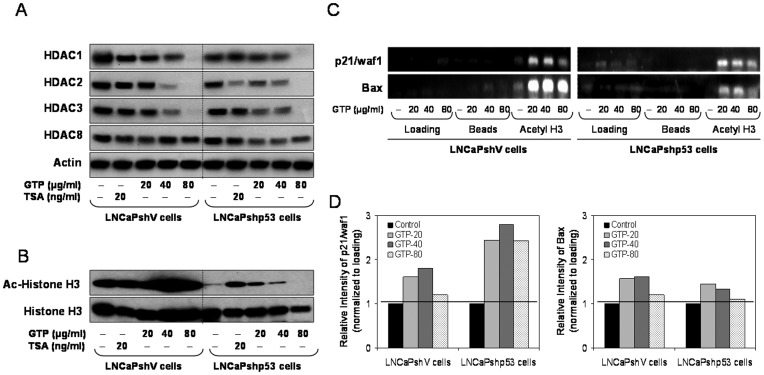
Green tea polyphenols suppresses class I HDAC expression, increases H3 histone acetylation and its binding to the p21/waf1 and Bax promoters in prostate cancer LNCaPshV and LNCaPshp53 cells. [**A**] Cells were exposed to 20-80 µg/ml concentration of GTP and 20 ng/ml of HDAC inhibitor TSA for 24 h and Western blotting was performed for HDAC 1, 2, 3 and 8. A typical actin blot demonstrates internal loading control. [**B**] Cells were treated with 20–80 µg/ml and 20 ng/ml of HDAC inhibitor, TSA for 24 h. Histones were acid extracted and Western blotting was performed for histones H3 and acetylated histones H3. Histone H3 was used as internal loading control. [**C**] Chromatin immunoprecipitation [ChIP] assay was performed for association of acetylated histone H3 with the promoters of p21/waf1 and Bax in these cells. [**D**] Relative intensities of acetylated histone H3 protein bands normalized to histone H3. The details are described in the materials and methods section.

We recently demonstrated that GTP suppresses class I HDAC in human prostate cancer cells [13], and the present study provides additional confirmation that GTP acts as an HDAC inhibitor. The HDAC expression was compared with TSA, which selectively inhibits class I and II HDACs. Firstly, we assessed the effect of p53 knockdown on the basal levels of class I HDAC expression. Compared to LNCaPshV cells, a significant decrease in the basal protein expression of HDAC1 and HDAC2 was observed in LNCaPshp53 cells, whereas expression of HDAC3 and HDAC8 remained unchanged in these cells (**Figure 5A**). Treatment with TSA decreased the expression of HDAC1 in LNCaPshV cells, whereas a marked decrease in HDAC2 expression was observed in LNCaPshp53 cells. GTP treatment decreased the levels of class I HDACs, particularly the levels of HDAC 1, 2 and 3, in a dose-dependent manner in both cell lines; however, a significant decrease was observed in LNCaPshp53 cells, compared to LNCaPshV cells (**Figure 5A**).

Next we determined whether decrease in HDAC expression affects histone acetylation. As shown in **figure 5B**, Western blot of acid extracted protein treated with varying doses of GTP exhibited a marked increase in histone H3 acetylation in LNCaPshV cells, compared to LNCaPshp53 cells. No change in the acetylation of histone H4 was observed in either cell line (data not shown). The increase in histone acetylation relaxes chromatin and enables binding of transcription factors to their promoters, leading to increased transcriptional activation of target genes.

### Green Tea Polyphenols Cause Differential Binding of Acetylated H3 to the Promoters of p21/waf1 and Bax Genes in LNCaPshV and LNCaPshp53 Cells

Next, we determined changes in the acetylation status of histone H3 associated with the promoter region of the p21/waf1 and Bax genes. Using anti-acetylated histone H3 antibody followed by PCR with the primers specific for p21/waf1 and Bax promoter, chromatin immunoprecipitation assay was performed. As shown in **figure 5C**, GTP treatment resulted in an increase in acetylated histone H3 associated with the p21/waf1 and Bax promoters in both cell lines, albeit at different levels. Binding of p21/waf1 and Bax to acetylated H3 was higher in LNCaPshV cells, compared to LNCaPshp53 cells (**Figure 5** **C&D**).

## Discussion

Prostate cancer is one of the major causes of cancer-related morbidity and mortality in males in the United States [30]. The p53 protein is a tumor suppressor gene product that can block the progression of cell division through the cell cycle and/or induce apoptosis. Loss of p53 function as a result of mutation or deletion in the p53 gene occurs in 24% of primary prostate tumors [31], [32]. Defects in the p53 pathway contribute to aggressive tumor behavior and are associated with chemoresistance, which remains a major hurdle in the treatment of prostate cancer [2]–[5]. It is therefore important to devise methods of inducing apoptosis in prostate cancer cells in the absence of functional p53. With this goal, we have previously demonstrated that green tea polyphenols (GTP) and their major constituent, epigallocatechin-3-gallate (EGCG), induce apoptosis in prostate cancer cells with variable p53 status [13]. However, the precise role of p53 inactivation and the involvement of molecular pathways in GTP-induced apoptosis have not been elucidated. To clarify these mechanisms, we studied two types of cells: LNCaP cell in which p53 was knocked out by transfection with shp53RNA in lentiviral vector and LNCaP cells modified by vector introduction. Our studies demonstrate that these two sets of isogenic prostate cancer cells with differing p53 status did not show any significant time course difference in GTP-mediated apoptosis. This report also presents substantial evidence that GTP-mediated apoptosis involves two independent pathways of cell death that include i) the extrinsic FAS-FADD death receptor pathway, and ii) the intrinsic pathway, with induction of activities in the mitochondria that initiate programmed cell death which differs with p53 status.

Our previous studies demonstrate that EGCG-mediated apoptosis stabilizes p53 by phosphorylation on critical serine residues in human prostate cancer LNCaP cells harboring wild type p53, suggesting that endogenous p53 is a critical determinant of EGCG-induced apoptosis [28]. Hastak *et al*. previously showed that reintroduction of wild-type p53 in mutant p53 prostate cancer PC-3 cells sensitized them to EGCG-mediated apoptosis. Furthermore their studies demonstrated that p53 inactivation in LNCaP cells using siRNA rendered these cells resistant to EGCG-mediated apoptosis [25]. Our results suggest that in contrast to mechanisms that occur in cells that harbor wild-type p53, p53-independent mechanisms dominate in the execution of GTP-induced cell death in prostate cancer cells lacking p53. In support of this premise, dose-dependent activation of p53 and its correlation with apoptosis was observed in LNCaPshV cells after GTP exposure; silencing of p53 gene expression did not alter GTP-mediated apoptosis in LNCaPshp53 cells in a time-dependent manner (**Figure 1** **A&C**). In addition, alterations in the protein expression of various genes that regulate cell survival and apoptosis after GTP exposure provided further clues as to its mode of action. In response to GTP treatment, LNCaPshV cells were arrested in G1 phase with a marked rise in p21/waf1 expression. Furthermore, the increase in p21/waf1 corroborates with increase in Bax in these cells. However, in LNCaPshp53 cells, an increased level of Bax and p21/waf1 was observed after GTP treatment, but not to the extent in LNCaPshV cells. The increased levels of p21/waf1 and Bax after GTP treatment in LNCaPshp53 cells was independent of p53 status. This confirms previous report suggesting that p21/waf1 can also be upregulated in a p53-independent manner [33]. Other p53 target genes such as PUMA also increased after GTP treatment in both cell lines, thereby attesting to a lack of p53-dependent effect. These results support the premise of p53-independent signaling, which may occur in the absence of functional p53 in GTP-mediated apoptosis.

It has been demonstrated that the extrinsic death receptor pathway represents a critical target in cancer chemotherapy. The extrinsic pathway is initiated upon receptor ligation (via FAS/CD95 ligand, TNFα or TRAIL) resulting in the assembly of the death-inducing signaling complex (DISC), in which procaspase-8 undergoes auto-proteolytic activation. There are reports regarding upregulation of FAS protein in cancer cells by various plant polyphenols, including EGCG, theaflavins, curcumin, and resveratrol [34]–[38]. Studies indicate that p38/MAPK and JNK/c-Jun amino-terminal kinases are putative kinases for FAS upregulation [34], [35]. Activation of FAS leads to upregulation of adapter protein, FADD through its phosphorylation [8]. Studies have demonstrated that phosphorylation of FADD at Ser194 can be induced by paclitaxel in human prostate cancer cells leading to cell cycle arrest and apoptosis [39]. In the present study, we demonstrate that GTP-induced JNK leads to activation of FAS, and FADD phosphorylation at Ser194 in both cell lines. Using a specific inhibitor of JNK, SP600125, GTP-mediated activation of JNK was significantly reduced, which correlated with p-FADD expression and extent of apoptosis. To the best of our knowledge, ours is the first study to demonstrate that green tea polyphenols cause FAS activation and phosphorylation of FADD through JNK in prostate cancer cells. A recent study showed that theaflavins (polyphenolic components of black tea) activate JNK and induce FAS, causing death of p53 mutated breast cancer cells [36]. We also clearly demonstrate that FAS activation occurs rapidly upon exposure to GTP, the use of FAS-neutralizing antibody does not alter GTP-induced apoptosis in these cells. Since inhibition of this pathway failed to completely block GTP-induced apoptosis, it is reasonable to infer the possibility of the involvement of alternate pathways as well.

Studies have demonstrated that Akt activation confers chemo-resistance and inhibits apoptosis in cancer cells, whereas suppression of Akt sensitizes the cells to chemotherapy-mediated apoptosis [40], [41]. The PI3K-Akt gene has been established as a major determinant for cell growth and survival in a wide range of human cancers [42]. Upregulation of PI3K-Akt signaling through mutations in the PTEN gene and constitutive activation of growth factor receptors leads to evasion of apoptosis in tumor cells [42]–[44]. Mutation and/or loss of function in the negative regulator PTEN has been observed in advanced stage human prostate cancers and in xenograft models [45], [46]. We have recently demonstrated the putative role of PI3K-Akt in prostate cancer progression in cell culture and in autochthonous transgenic adenocarcinoma of the mouse prostate [47]. In nearly 50% of prostate cancers, the PI3K-Akt survival pathway has been shown to be constitutively upregulated because of loss of function and/or mutation of tumor suppressor PTEN, which functions as a negative regulator of PI3K through its lipid phosphatase activity [48]. In addition, the PI3K-Akt pathway has been implicated in chemo- and radiation- resistance [49], [50]. Therefore, agents that inactivate the PI3K-Akt-signaling pathway are worthy of investigation for their use in treating prostate cancers, particularly those with aggressive biologic behavior related to defects in p53 function and signaling.

In our studies, we demonstrate that knockdown of p53 results in Akt activation and increase in BAD phosphorylation in LNCaPshp53 cells. Altered Akt/BAD signaling contributes to mitochondrial dysfunction and logically points towards the possible involvement of Akt-associated pathways in GTP-induced cancer cell apoptosis via mitochondrial death cascade. Our studies demonstrate that GTP treatment causes a marked decrease in activating phosphorylation of Akt at Ser473 in LNCaPshp53 cells. The GTP-mediated suppression of Akt phosphorylation in these cells is coupled with inhibition of BAD phosphorylation, an essential downstream target of the Akt-signaling pathway. Activated Akt can phosphorylate several apoptosis-regulating proteins, including the proapoptotic Bcl-2 family protein, BAD [42], [43]. BAD promotes cell death by interacting with anti-apoptotic Bcl-2 members such as Bcl-_xL_, which allows the multi-domain proapoptotic Bcl-2 family members Bax and Bak to aggregate and cause release of apoptogenic molecules (e.g. Cytochrome c) from mitochondria to the cytosol, culminating into caspase activation and cell death [51]. BAD is phosphorylated at Ser136 by Akt, reducing its ability to interact with Bcl-_xL_ [52]. Our study indicates that GTP-mediated inactivation of Akt is associated with reduced phosphorylation of BAD at Ser136 in LNCaPshp53 cells. Because the PI3K-Akt/pBAD pathway has emerged as an important regulator of cancer cell survival, agents with direct or indirect effect on the Akt pathway are logical choices for development as new therapeutic tools for managing prostate cancer. Sustained inhibition of Akt-BAD phosphorylation has been reported in prostate cancer cells by quercetin and apigenin [53], [54]. Importantly, inhibition of the Akt survival pathway with inactivated p53 was shown in further experiments in which Akt was inhibited by PI3K inhibitor LY294002. This experiment demonstrated that inhibition of Akt by LY294002 resulted in loss of survival in both cell lines, an effect that also occurred upon exposure to GTP. It was shown that the combination of LY294002 with GTP further increased apoptosis in cancer cells, particularly in LNCaPshV cells, confirming the independent involvement of Akt/BAD pathways in GTP-induced apoptosis of p53 inactivated prostate cancer cells.

Having clarified the two pathways of GTP-mediated apoptosis, we turned our attention to downstream events. It has been reported that the cleavage of the BH3 domain only protein BID (BH3 interacting death agonist) and non-phosphorylated BAD form heterodimers with Bcl2 family cell death suppressors and execute programmed cell death involving the loss of mitochondrial membrane potential through cytochrome C release in the cytosol [51]. Simultaneously, cleavage of BID by caspase 8, the initiator caspase for the extrinsic pathway, results from the formation of truncated BID (tBID), enhancing pore formation in the mitochondrial membrane. Crosstalk between the extrinsic and intrinsic apoptotic pathways has been reported by others [36]. In our studies, GTP-induced activation of caspase 9 indicates the possibility of crosstalk between the two pathways. Both these pathways converge during downstream signaling, resulting in tBID. We demonstrated cleavage of caspase 3 and release of cytochrome c from mitochondria to the cytosol as early as 6 h after initiation of GTP exposure in both cell lines, consistent with the premise of independent involvement of these two pathways, converging at the execution phase in both p53-dependent and p53-independent manner.

The stability and transcriptional activity of p53 are regulated by posttranslational modifications, including phosphorylation and acetylation [55]. We have previously demonstrated that GTP and EGCG induce apoptosis in prostate cancer LNCaP cells through p53 stabilization by phosphorylation on several critical serine residues [28]. More recently, we have demonstrated that GTP and EGCG activate p53 through acetylation at the Lys373 and Lys382 residues by inhibiting class I HDACs in human prostate cancer LNCaP cells [12]. The increased p53 acetylation augments its binding on the promoters of p21/waf1 and Bax, thereby promoting cell cycle arrest and apoptosis. The present study demonstrates that GTP exposure suppresses class I HDACs regardless of p53 status, possibly resulting in the acetylation of wild type p53, increasing its half-life and its binding to the p21/waf1 and Bax promoter. Reports suggest that p300/CBP, the histone acetyltransferase, potentiates p53-dependent transcription activation, and that acetylation of p53 by p300 markedly stimulates its sequence-specific DNA binding and transcriptional activity. This synergistic enhancement of p53 transactivation activity is a result of at least two different pathways, including acetylation of core histone proteins and p53 acetylation, effects that are readily demonstrated in LNCaPshV cells that are exposed to GTP. In the present study, GTP-induced acetylation of histone H3 was more pronounced in LNCaPshV cells, and this effect was associated with increased binding to the p21/waf1 and Bax promoter, compared to LNCaPshp53 cells. In addition, other p53-independent cellular events such as downregulation of c-IAP and XIAP may be further required for GTP-induced apoptosis.

Most studies indicate that the frequency of p53 mutations is higher in advanced-stage prostate cancers [32], and that such cancers are relatively resistant to chemotherapeutically-induced apoptosis [56]. In contrast, our studies demonstrate that GTP exposure induces apoptosis in prostate cancer cells with or without functional p53, and that two independent but parallel pathways mediate apoptosis in prostate cancer cells, merging with one another through the mitochondrial death cascade. Although some investigators have suggested that the exposure levels to GTP used in cell culture studies could not be realistically achievable *in* *vivo* because of rapid metabolism and poor absorption [57], others have shown that the bioavailability of polyphenols is different in various types of tissue [58], indicating the possibility that they may be efficacious in the prevention and/or treatment of cancer.

### Conclusions

**Figure 6:**
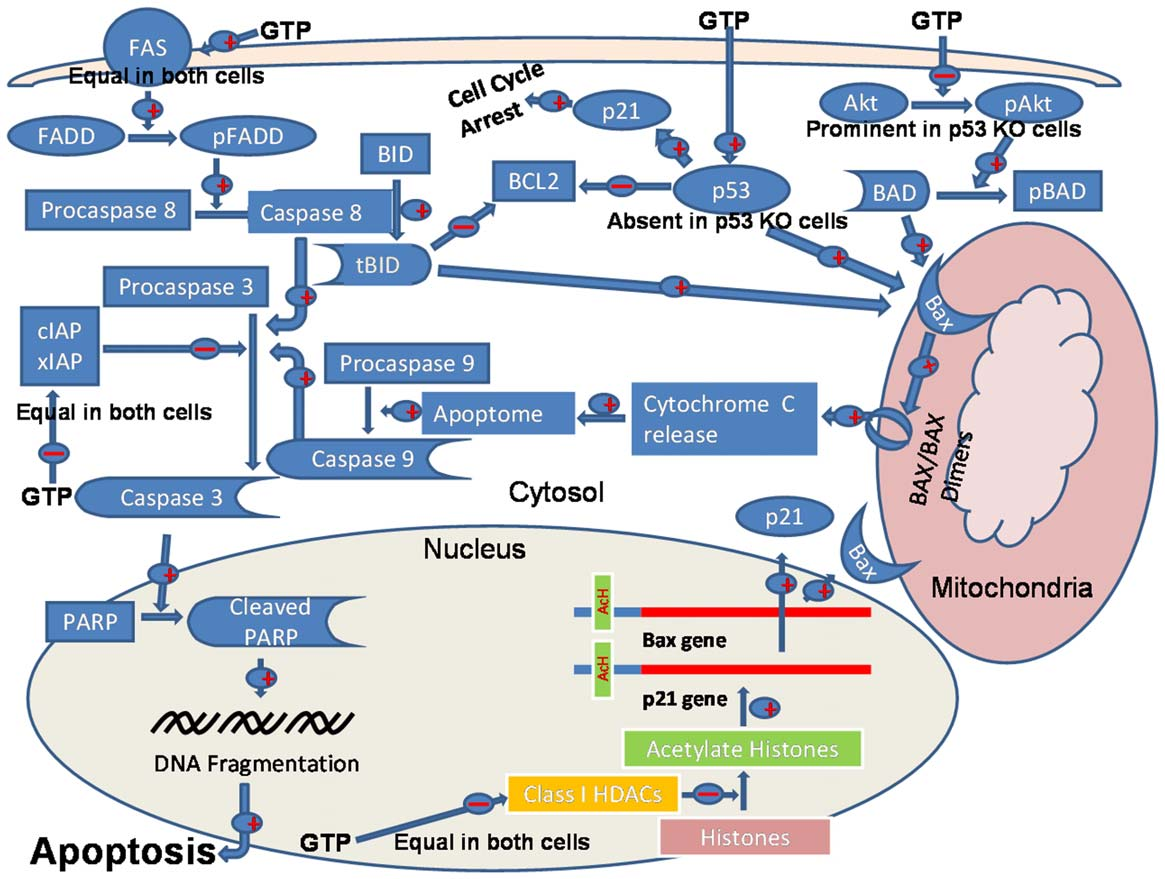
Proposed model of the molecular mechanism of green tea polyphenols-induced activation of extrinsic (death receptor pathway) and intrinsic (mitochondrial death cascade) in the presence and absence of p53. GTP has shown to affect epigenetic and various signaling pathways. GTP induces activation of extrinsic pathway of apoptosis to similar extent irrespective of their p53 status. However, intrinsic pathway is activated by p53 upregulation in cells possessing wild-type p53; whereas it is activated by inhibition of Akt, which in turn dephosphorylate BAD and activate intrinsic pathway in the cells lacking p53. The GTP-mediated cell cycle arrest and eventually cell death via induction of apoptosis is caused by upregulation of p21/waf1 due to downregulation of class-I HDACs, which in turn causes increase acetylation of histone H3 and its binding on to the promoter of p21/waf1, leading to increased p21/waf1expression in prostate cancer LNCaP cells irrespective of their p53 status. 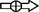 demonstrate activation and 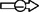 demonstrate inhibition.

In summary, we found that green tea polyphenols effectively promote apoptosis in cancer cells both in the presence or absence of p53 function, through perturbations in signaling pathways, including the extrinsic death receptor FAS-FADD and survival pathways that converge in the execution of apoptosis through involvement of the mitochondrial death cascade (**Proposed model;** **Figure 6**). These results, in combination with epidemiological data and data from interventional studies, provide strong support for further exploration of the role of green tea polyphenols in chemoprevention of prostate cancer and as therapeutic agents for established prostate cancer, particularly those cancers that have become unresponsive to current conventional treatments.
